# Magnetic Properties of SmCo_5_ + 10 wt% Fe Exchange-Coupled Nanocomposites Produced from Recycled SmCo_5_

**DOI:** 10.3390/nano10071308

**Published:** 2020-07-03

**Authors:** Arnab Chakraborty, Răzvan Hirian, Gregor Kapun, Viorel Pop

**Affiliations:** 1 Research and Development, MAGNETI Ljubljana d.d., 1000 Ljubljana, Slovenia; arnab.chakraborty@etn-demeter.eu; 2 Jožef Stefan International Postgraduate School, 1000 Ljubljana, Slovenia; 3 Faculty of Physics, Babeş-Bolyai University, 400084 Cluj-Napoca, Romania; viorel.pop@phys.ubbcluj.ro; 4 Department for Material Chemistry, National Institute of Chemistry, 1000 Ljubljana, Slovenia; Gregor.Kapun@ki.si

**Keywords:** soft/hard magnetic nanocomposites, recycled magnets, interphase exchange coupling, mechanical milling

## Abstract

Nanostructured alloy powders of SmCo_5_ + 10 wt% Fe obtained using recycled material were studied for the first time. The SmCo_5_ precursor was obtained from commercial magnets recycled by hydrogen decrepitation. The results were compared with identically processed samples obtained using virgin SmCo_5_ raw material. The samples were synthesized by dry high-energy ball-milling and subsequent heat treatment. Robust soft/hard exchange coupling was observed—with large coercivity, which is essential for commercial permanent magnets. The obtained energy products for the recycled material fall between 80% and 95% of those obtained when using virgin SmCo_5_, depending on milling and annealing times. These results further offer viability of recycling and sustainability in production. These powders and processes are therefore candidates for the next generation of specialized and nanostructured exchange-coupled bulk industrial magnets.

## 1. Introduction

Permanent magnets (PMs) are the drivers of modern technology and are crucial to industry. Modern PMs are intermetallic alloys containing appreciable amounts of lanthanide elements alongside iron and cobalt [1,2,3,4]. Except Fe, the other constituent elements come from minerals mined in regions of conflict or are susceptible to geopolitical control. The supply risks compounded with the high environmental and human costs drive us to novel PM [1,5,6]. Soft/hard interphase exchange-coupled magnets (spring magnets) offer a way to minimize dependence on such critical minerals while still providing high performance [7,8].

Exchange-coupled nanocomposite magnets consist of soft and hard magnetic phases in a diffuse nano-scale composite, coupled by interphase exchange coupling. In exchange-coupled nanocomposites (ECNs), exchange coupling between neighboring soft and hard magnetic crystallites improves the energy product, *(BH)_max_*, when the structure and microstructure meet certain material-dependent criteria [8,9,10,11,12]. Theory has been refined by micromagnetic simulations, which place critical limits on the material design of ECN; namely the critical dimension of the soft magnetic inclusions, uniform granularity, and volume fraction (capped below 40% to maintain a high *(BH)_max_*) [11,13]. Experiments confirm that a high soft magnetic volume fraction dramatically reduces the coercivity [14,15,16,17]. Both nucleation of reverse domains and their propagation increase in such a case, because the system begins to behave as a soft magnetic bulk doped with a hard phase [7,16,17,18,19,20].

The biggest promises of exchange-coupled nanocomposites are miniaturization (due to the increased *(BH)_max_*) and sustainability by the reduction of the rare earth mass fraction (10–20% reduction) and efficiency increase. In this work, we explore the possibility of improving on the sustainability aspect by creating exchange-coupled nanocomposites from recycled SmCo_5_ magnets [21,22,23] (90 wt%) and a cheap and available 3d metal (Fe, 10 wt%). While most of the recent work in this area is on Nd_2_Fe_14_B+Fe nanocomposites [24,25,26,27,28], the choice of recycled SmCo_5_ was made due to the relative purity of these industrial magnets compared with other compositions [2,3]. These types of materials could minimize purchase and environmental costs due to critical elements, adding to the savings accrued by enhanced magnetic properties.

## 2. Materials and Methods

Our material choice is dictated by the naturally large saturation magnetization available in Fe: *M_s_* ≈ 215 Am^2^/kg and the high intrinsic anisotropy of SmCo_5_: *K_1_* ≈ 17.2 MJ/m^3^. The SmCo_5_, (production supply; MAGNETI Ljubljana d.d., Slovenia) used was either virgin (jet-milled particles <40 μm), or recycled from production magnets by hydrogen decrepitation (coarse particles ~200 μm) [29]. The Fe used was produced by inert gas atomization (size <40 μm; Högnäs AB, Sweden). All materials were handled under the protected argon atmosphere of a dry glovebox (MB100; <5 ppm O_2_; MBraun, Garching bei München, Germany). Mechanical hardness of all precursor materials was tested by the Vickers micro-indentation method (Type-M 4960; 10 s; Shimadzu, Kyoto, Japan) using 1 N for SmCo_5_ and 0.15 N force for Fe.

Dry high-energy ball-milling (dHEBM)Vario-Planetary Mill PULVERISETTE 4 classic line; Fritsch, Idar-Oberstein, Germany) was used to produce a nano-disperse powder of compositional choice: SmCo_5_ + 10 wt% Fe. Mechanical milling (MM) was done in stainless steel vials (80 mL; Fritsch, Idar-Oberstein, Germany), sealed under Ar with 440 C stainless steel balls (10 mm diameter; 107 g total mass) for durations: 2, 4, and 6 h. The powder-to-ball mass ratio was 0.1, and planetary-to-sun wheel rotation ratio -900/333. This latter ratio is based on prior work that shows low-friction milling as optimum for the desired microstructure [30]. Every 2 h, the milling vials were opened in the glove box—to scrape out the milled product, grind it in a ceramic mortar and pestle, and recommence milling. This helped collect samples at 2 and 4 h while also promoting homogenization. Using scanning electron microscopy (SEM) (Ultra Plus; ZEISS, Oberkochen, Germany) at acceleration voltages between 1.2 and 1.5 kV, the size and morphology of the resulting milled products were checked.

To relax internal stresses and recrystallize the hard phase, heat treatment was necessary. Temperature/time pairs: 420 °C for 8 h, 510 °C for 0.5 h, 510 °C for 8 h, and 600 °C for 0.5 h were selected for heat treatment under dynamic vacuum (≈10^−9^ bar) in quartz tubes. The oven was pre-heated, milled products were packed in tantalum-foil capsules during the process, and cooling was in furnace.

Duration of milling was guided by prior work in the lab and existing literature [16,17,18,19,20,31,32]. The temperature range for heat treatment was selected to compensate for the disorder introduced during milling, while aiming for optimum relaxation and recrystallization of the milled products. The annealing conditions of the milled products were determined by differential scanning calorimetry (DSC) (STA-Q600; TA Instrument, New Castle, DE, USA) under Ar atmosphere at a heating rate of 20 °C per minute. The structure and phase evolution of the samples were studied by X-ray diffraction (XRD) using D8 Advance diffractometer (Bruker, Germany) equipped with Cu Kα source. Using Scherrer’s equation, the average crystallite sizes for the SmCo_5_ phase (peak at 30.48°) were estimated [33].

Magnetic characterization was carried out on the powder fixed in epoxy, using a vibrating sample magnetometer (Cryogenics, London, UK) in magnetic field of ±10 T at 300 K. The derivative of magnetization (*dM/dH*) was also computed from demagnetisation curves. As the estimation of the powder mass inside the epoxy matrix is susceptible to errors, the value of the magnetization, *M,* in the demagnetization curves was calibrated to the magnetization value at 10 T measured for free magnetic powder, for which the mass could be accurately measured as:

(1) $$M{\left(H\right)}^{cal.\;}=M{\left(H\right)}^{epoxy}\times M_{\left(10T\right)}^{free}/M_{\left(10T\right)}^{epoxy}.$$

Here, *M^cal^* is the calibrated magnetization (used in the plots shown in this work), *M^epoxy^* is the recorded magnetization of the powder blocked in epoxy, and *M^free^* is the magnetization of the free magnetic powder.

The saturation magnetization *M_s_* for the studied isotropic nanocomposites was determined using the approach to the saturation law [34]:

(2) $$M\left(H\right)=M_{s}\left(1-a_{1}/H-a_{2}/H^{2}\right)+\chi H$$

where *H* is the value of the magnetic field and *a_1_* and *a_2_* are coefficients that describe the low- and high-field part of the magnetization curve, respectively, and *χ* is the paramagnetic-like factor at the high field.

## 3. Results and Discussions

The mechanical milling for 2 h and the heat treatment at 420 °C do not result in important changes of the starting mixed powders. Consequently, we decided not to discuss these results. However, the XRD data for the nanocomposites milled for 4 h and 6 h and annealed at 420 °C, along with the measured demagnetization curves and *dM/dH* plots are given in Figures S1, S2 and S3 respectively, available in the Supplementary Information section. These materials perform poorly due to the fact that the hard magnetic phase is not recrystallized, and therefore are not discussed in the paper.

**Figure 1:**
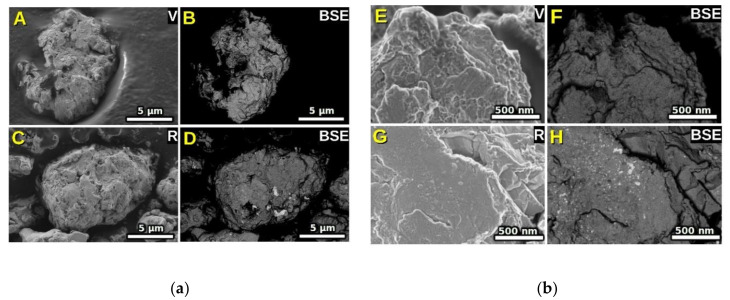
(**a**) SEM images at a scale of 5 μm of SmCo_5_ + 10 wt% Fe nanocomposite powders made using virgin (V) SmCo_5_ imaged by secondary electrons (Panel A) or backscattered electrons (Panel B) and recycled (R) SmCo_5_ imaged by secondary electrons (Panel C) and backscattered electrons (Panel D). (**b**) higher magnification images at a scale of 500 nm; Panels E, F, G and H are higher magnification images of A, B, C and D respectively.

The SEM investigation of the products of 6-h milling is presented in Figure 1. The secondary electron (SE) micrographs, A (virgin) and C (recycled) SmCo_5_ + 10 wt% Fe nanocomposite powders, Figure 1a, show very compacted high-aspect ratio flakes in all cases. Even at higher magnifications, Figure 1b, we notice no conspicuous un-milled large particles—and therefore speculate an even dispersion. The respective backscatter electron (BSE) micrographs, B (virgin) and D (recycled) nanocomposites, Figure 1a, show similar homogeneity without any appreciable features. Panel C also shows large particles of a broad range of sizes, usual for dry milling. A difference in the surface morphology, between the virgin and recycled milled products, is also evident at high magnification in panels E to H, Figure 1b. The white regions observed in the BSE images, panels D and H, Figure 1, denote some traces of Sm oxides in the recycled samples. These spots are absent in the virgin material (panels B and F, Figure 1).

**Figure 2:**
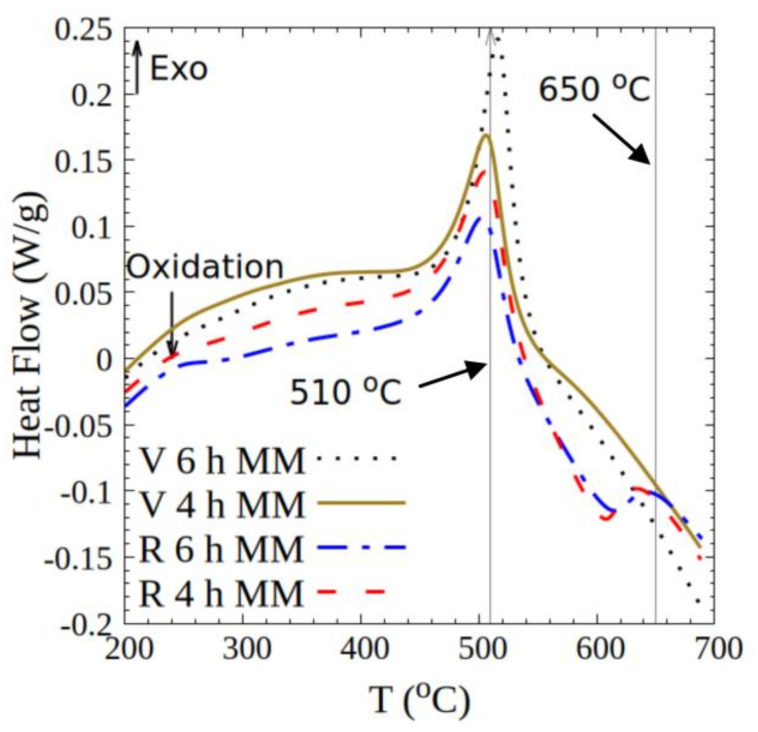
Differential scanning calorimetry (DSC) plots for 4 and 6 h mechanically milled (MM) virgin (V) and recycled (R) samples.

DSC measurements of the milled products, Figure 2, show the exothermic plateau of stress relaxation at temperatures below 400 °C. The recrystallization signal typical of pure iron, in the range between 400 and 500 °C, is convoluted with the recrystallization of SmCo_5_ at 500 °C [35]. The exothermic peak between 620 and 650 °C can be attributed the phase transition from SmCo_5_ + Fe → Sm_2_(Co_1−*x*_Fe*_x_*)_17_ + SmCo_5_, [17,36]. Some free cobalt could be formed through the oxidation of Sm as SmCo_5_ + O_2_ → Sm_2_O_3_ + Co. This Co should also be involved in the formation of the 2:17 phase and may enter the structure of Fe [18,37]. Both Sm_2_O_3_ and Sm_2_(Co_1−*x*_Fe*_x_*)_17_ are detrimental to the ECNs. The latter is detrimental due to the low pinning and nucleation fields between Fe and the 2:17 phase [37,38]. Both the virgin and recycled materials show the expected features, with peculiar differences in enthalpy signatures [39]. The virgin material is far more impacted by 6 h milling—showing a larger amorphous fraction, denoted by the very high relative intensity of the exothermic peak at approximately 500 °C. This difference in the DSC curves of virgin and recycled materials results from the differences in their mechanical properties (discussed further below).

We posited (post-hoc) that the virgin raw material is mechanically different from the recycled raw material due to the process they each undergo in their production. While the recycled material is obtained from the decrepitation of hard-sintered magnets undergoing mechanical agitation under high hydrogen pressure [29], the virgin material is instead a product of jet milling. Jet milling involves high-energy comminution to fine particles in the size range of tens of microns. The process introduces tremendous stresses and a large number of dislocations in the material, governed by Rittinger’s law (valid below ~100 µm) [40]. This causes the virgin raw material to be harder and more brittle but less ductile. During dHEBM, fracture and comminution are initially privileged over plastic deformation—creating smaller particles of virgin SmCo_5_. The mechanical differences are confirmed by micro-indentation hardness testing on the starting materials, which show that the Vickers hardness is *H_v_* = 702*HV*0.1 for the virgin SmCo_5_ precursor; whereas for the recycled SmCo_5_ precursor, *H_v_* = 620*HV*0.1 as expected (the Fe precursor has *H_v_* = 85*HV*0.015). Additionally, this validates our experience during scraping out the milled product every two hours, where we found that the virgin material would agglomerate less and was easily pulverized relative to its recycled counterpart. In every milling batch, agglomeration progressively increased for both materials, from 4 to 6 h. We further extend this correlation with X-ray diffraction data and measured magnetic properties.

**Figure 3:**
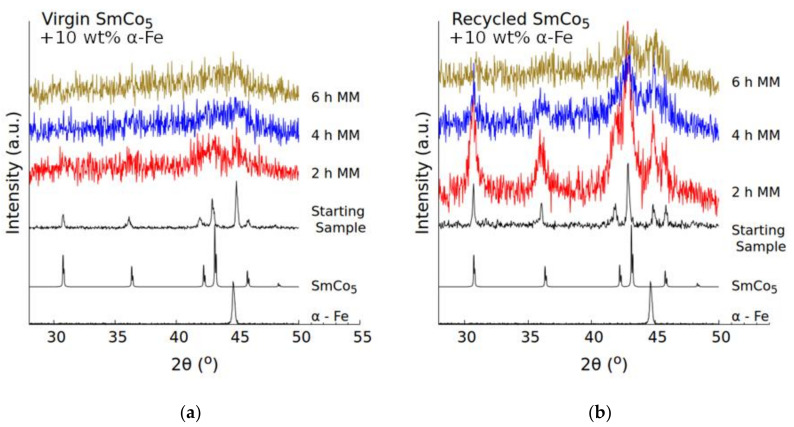
XRD patterns for starting mixture and as-milled (2, 4, and 6 h) virgin (**a**), and recycled materials (**b**). Primary peaks for SmCo_5_ and Fe have been marked.

The diffraction patterns for the as-milled samples, Figure 3, show that the structure of the hard and soft magnetic phases becomes progressively damaged as milling time is increased. These measurements are coherent with the hardness measurements. The increased hardness of the virgin material leads to a much quicker amorphization of both the SmCo_5_ and Fe structures. For the samples made using virgin SmCo_5_, Figure 3a, after 2 h MM, most long-range order is destroyed, and only two broad humps are visible where the most intense peaks of the two phases should be. After 6 h of milling, they present only a barely visible hump around 45°. By contrast, for the materials made using recycled SmCo_5_ (Figure 3b) at 2 h MM, all the peaks of the two phases are clearly visible. Moreover, even after 6 h of MM, the diffraction peaks of the SmCo_5_ and Fe phases can still be identified (for the samples made using recycled material), even if they are quite broad.

**Figure 4:**
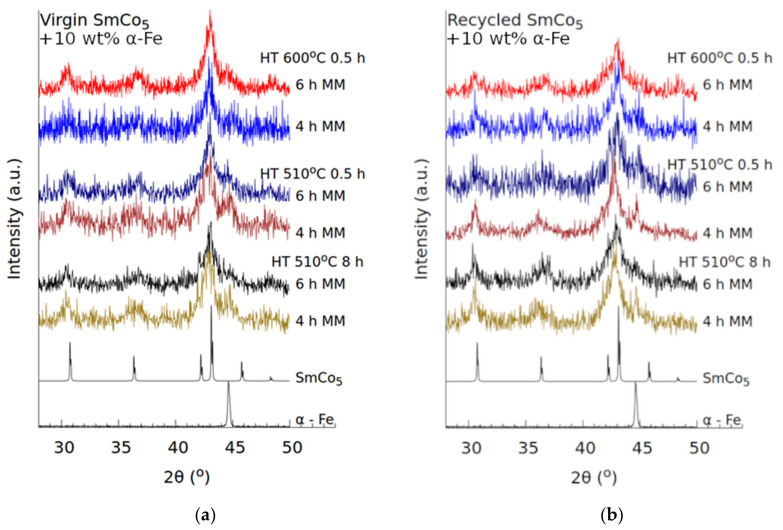
XRD patterns for (**a**) virgin and (**b**) recycled materials after heat treatment (HT). Primary primary peaks for SmCo_5_ and Fe have been marked.

The XRD patterns for the annealed samples, Figure 4, show peak broadening associated with small crystallites and structural damage generally associated with dHEBM. In general, samples milled for 4 h show a higher degree of definition for the Fe and SmCo_5_ peaks when compared with 6 h milling. This is only natural, as these samples have fewer defects, due to the lower milling time.

The XRD study shows that heat treatment improves the crystallinity—which is reflected in the sharpening of the major peaks associated with SmCo_5_ and Fe and reduction in their full-width at half-maximum (FWHM). The low signal-to-noise ratio of the diffraction patterns makes quantitative conclusions difficult. We do not observe prominent peak signatures (compared with background) for Sm_2_Co_7_, SmCo_3_, Sm_2_(Co,Fe)_17_, or Sm_2_O_3_—and cautiously conjecture low volume fractions for the same. It should be noted that formation of intermediate Sm–Co–Fe phases (due to alloying) is common during dHEBM. When limited to the interface, this compositional gradient is considered beneficial to interphase exchange coupling [18,32,41,42]. While the crystallite sizes for the soft magnetic phase could not be determined, the estimated crystallite sizes for the SmCo_5_ phase are given in Table 1. The analysis shows that, in all cases, the hard magnetic phase crystallites grow with annealing time and temperature, as is expected. The crystallite sizes are fairly consistent across samples, annealing for half an hour, yielding average values between 7 and 10 nm at 510 °C and between 12 and 20 nm at 600 °C.

The interphase exchange coupling depends on the structure and microstructure, which is in turn determined by the duration of milling. Longer milling durations lead to finer and more even dispersion of Fe in the resulting nanocomposite and lead to better coupling, whereas the annealing is responsible for recovery of structure, which positively impacts *M_r_* and *μ_0_H_C_*.

**Figure 5:**
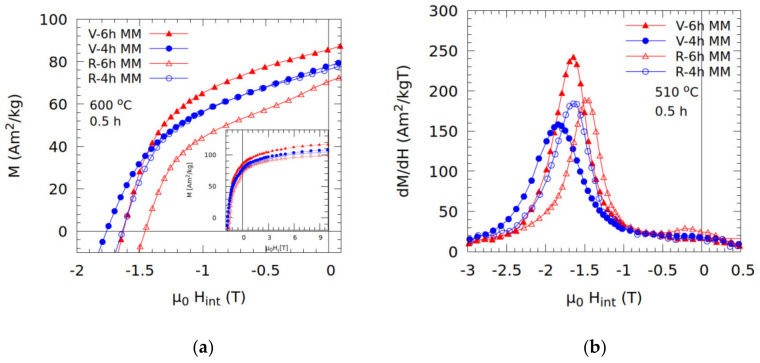
The demagnetization curves (**a**) as well as the *dM/dH* curves (**b**) for virgin (V) and recycled (R) samples milled for 4 and 6 h and annealed at 600 °C for 0.5 h. Magnetization curves, up to 10 T, for the samples are given in the figure (a) inset.

The demagnetization curves and *dM/dH* plots for virgin and recycled samples annealed at 600 °C for 0.5 h are presented in Figure 5a,b, respectively. The absence of major kinks in the demagnetization curves demonstrates robust coupling. Of note is the exceptional coercivity shown by the virgin sample milled for 4 h, *μ_0_H* ≈ 1.76 T (and *M_r_* ≈ 77 Am^2^/kg)—the highest of all tested samples. The recycled sample milled for 4 h shows a high *μ_0_H_C_* ≈ 1.64 T (*M_r_* ≈ 76 Am^2^/kg). Milling for 4 h is also a factor in high coercivity—we conjecture, due to a less-damaged structure. The recycled sample milled for 6 h has reduced *M_r_* and *H_c_* values and shows mild decoupling. This is presented as a kink below field *μ_0_H_int_* ≈ 0.5 T perceptible in the *dM/dH* plot. By contrast, the virgin counterpart milled for 6 h shows excellent properties: *M_r_* ≈ 85 Am^2^/kg and *μ_0_H_C_* ≈ 1.63 T. This is again in line with the structure and microstructure resulting from the mechanical property differences we found.

**Figure 6:**
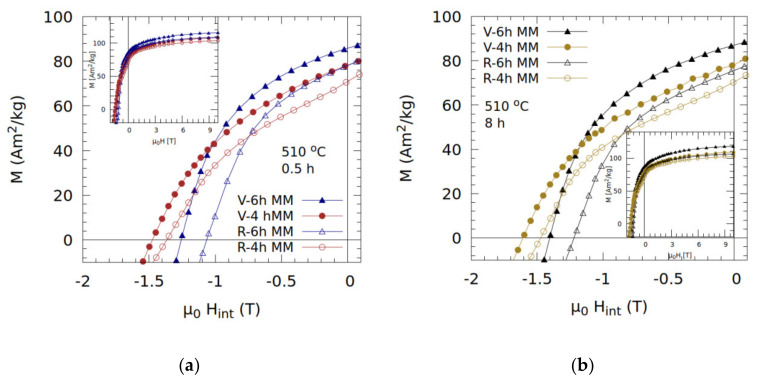
The demagnetization curves for virgin (V) and recycled (R) samples milled for 4 or 6 h and annealed at 510 °C (**a**) for 0.5 h and (**b**) 8 h. Magnetization curves, up to 10 T, for the respective samples are given in the figure insets.

The demagnetization curves for samples annealed at 510 °C for 0.5 and 8 h are given in Figure 6a,b, respectively. In both cases, the curves are very smooth, indicative of good interphase exchange. The heat treatments result in better coercivities for 4-h milled samples, while additional milling (6 h) improves remanence at the expense of coercivity, which indicates an improvement in interphase exchange with increased milling time.

**Figure 7:**
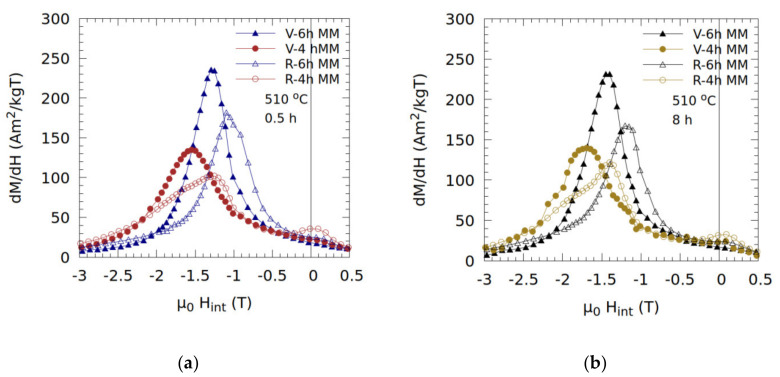
*dM/dH* vs. *H* plots for virgin (V) and recycled (R) samples milled for 4 or 6 h and annealed at 510 °C (**a**) for 0.5 h and (**b**) 8 h.

The *dM/dH* vs. *H* plots are given in Figure 7a,b for the samples annealed at 510 °C. These plots show that the interphase exchange coupling improves with milling time (up to 6 h) for both the virgin and recycled samples. The poor coupling for the 4 h MM samples is well illustrated by the presence of multiple peaks in the *dM/dH* vs. *H* curves, Figure 7a and b: (i) a peak at small fields, corresponding to a non-coupled soft magnetic phase; (ii) a high peak around *H_c_*, corresponding to the exchange-coupled composite, and (iii) a shoulder at even higher magnetic fields corresponding to non-coupled the hard magnetic phase.

The highest coercivity is noted in the virgin sample milled for 4 h and annealed at 510 °C for 8 h, with *μ_0_H_C_* ≈ 1.61 T (*M_r_* ≈ 78 Am^2^/kg). The virgin sample milled for 6 h and annealed at 510 °C for 8 h, shows exceptional *M_r_* ≈ 87 Am^2^/kg—the highest of all tested samples (and *μ_0_H_C_* ≈ 1.4 T). The shorter heat treatment for 0.5 h at 510 °C produces samples with reduced exchange coupling compared with longer heat treatment for 8 h. By contrast, milling for 6 h lowers coercivity and improves the achievable remanence, which is a sign of improved interphase exchange coupling.

The magnetic properties of studied samples are summarized in Table 2. The *M_S_* values (inset of Figure 6a) for virgin samples are found to be approximately 120 Am^2^/kg, and the *M_S_* values for recycled samples were close to 110 Am^2^/kg. This behavior can be explained by the small differences in phase compositions as we found in Figure 1D or 1H, where some oxides were observed in recycled samples. The diminution of the *M_s_* and *M_r_* values, by increasing annealing temperature or times, for recycled samples milled for 6 h can be explained by the insertion of Fe into the 2:17-type structure. The evolution of *M_r_/M_s_* ratio shows that interphase exchange coupling is improved by higher milling times, and values between 0.66 and 0.75 show good to very good interphase exchange coupling in studied samples. By contrast, using an estimated density (from component structures and phase fractions) for the nanocomposite powders, we arrive at the computed *(BH)_max_* up to 145 kJ/m^3^ for this magnetic system.

## 4. Conclusions

In this study, we report highly exchange-coupled SmCo_5_ + 10 wt% Fe nanocomposites produced from recycled SmCo_5_ magnets that perform comparably with virgin precursors. We confirm that high magnetization and good coercivity can be achieved with a top-down process, such as mechanical milling, in conjunction with well-planned heat treatment. The best obtained magnetic properties of magnetic nanocomposites using recycled hard magnetic phase are promising: *M_r_* = 78 Am^2^/kg, *μ_0_H_C_* = 1.64 T, *(BH)_max_ =* 117 kJ/m^3^ and *M_r_/M_s_* = 0.71. These values are 80–97% of the best values obtained in samples where we used virgin hard magnetic phase. Moreover, in the case of samples milled for 4 h and annealed 600 °C, the *(BH)_max_* obtained for the recycled material is 95% of the value obtained for the virgin composition. Further enhancement in magnetic properties could be obtained by optimizing the microstructure of the samples, by improving the recovery of the hard magnetic phase from magnets, or lowering its mechanical hardness to facilitate the formation of the composite during mechanical milling. Therefore, we conclude that the production of exchange-coupled magnetic nanocomposites via the recycling of permanent magnets may pave the way for an additional sustainable production route for permanent magnets, but the mechanical properties of the powder mixtures must be improved to overcome the limitations in their production.

## Figures and Tables

**Table 1:** Estimated crystallite sizes for the hard magnetic phase in SmCo_5_ + 10 wt% Fe nanocomposites.

Material	Milling Duration	Heat Treatment		FWHM	FWHM Error	Crystallite Size	Size Error
		Temp.	Duration	(deg)	(deg)	(nm)	(nm)
Virgin SmCo_5_ + 10 wt% Fe	6 h	600 °C	0.5 h	0.7	0.1	13	1
		510 °C	8 h	0.7	0.2	12	5
			0.5 h	0.8	0.2	10	3
	4 h	600 °C	0.5 h	0.6	0.1	14	2
		510 °C	8 h	0.8	0.1	11	2
			0.5 h	1.1	0.2	7	1
Recycled SmCo_5_ + 10 wt% Fe	6 h	600 °C	0.5 h	0.5	0.1	18	3
		510 °C	8 h	1.0	0.4	8	3
			0.5 h	0.9	0.3	9	3
	4 h	600 °C	0.5 h	0.4	0.2	21	8
		510 °C	8 h	0.7	0.2	12	4
			0.5 h	1.1	0.2	8	1

**Table 2:** Magnetic properties of 4 and 6h MM samples annealed at 510 and 600 °C. Uncertainties in *M_s_* ≈ 1%, *M_r_* ≈ 0.2%, and in *(BH)_max_* ≈ 3%.

Material	Milling Duration	Heat Treatment		*M_s_*	*M_r_*	*M_r_*/*M_s_*	*μ_0_H_C_*	*(BH)_max_*
		Temp.	Duration	(Am^2^/kg)	(Am^2^/kg)		(T)	(kJ/m^3^)
Virgin SmCo_5_ + 10 wt% Fe	6 h	600 °C	0.5 h	114	85.6	0.75	1.63	140.9
		510 °C	8 h	119	87.0	0.73	1.40	145.5
			0.5 h	118	85.9	0.73	1.26	141.5
	4 h	600 °C	0.5 h	118	77.9	0.66	1.76	116.5
		510 °C	8 h	108	78.2	0.72	1.61	117.4
			0.5 h	109	78.3	0.72	1.48	117.6
Recycled SmCo_5_ + 10 wt% Fe	6 h	600 °C	0.5 h	106	70.5	0.67	1.46	95.5
		510 °C	8 h	110	75.6	0.69	1.22	109.6
			0.5 h	112	78.1	0.70	1.07	117.1
	4 h	600 °C	0.5 h	108	76.2	0.71	1.64	111.5
		510 °C	8 h	105	70.4	0.67	1.47	95.2
			0.5 h	107	71.0	0.66	1.37	96.7

## Supplementary Materials

The following are available online at https://www.mdpi.com/2079-4991/10/7/1308/s1, Figure S1: XRD patterns for SmCo_5_ + 10 wt.%Fe magnetic nanocomposite, made using virgin or recycled SmCo_5_, milled for 2, 4 and 6 h and annealed at 420 °C for 8 h; Figure S2: Demagnetization curves (recorded at 300 K) for SmCo_5_ + 10 wt.%Fe magnetic nanocomposite, made using virgin (V) or recycled (R) SmCo_5_, milled for 4 and 6 h and annealed at 420 °C for 8 h. Magnetization curves up to 10 T for the respective samples are given in the inset; Figure S3: *dM/dH* vs *H* plots (recorded at 300 K) for SmCo_5_ + 10 wt.%Fe magnetic nanocomposite, made using virgin (V) or recycled (R) SmCo_5_, milled for 4 and 6 h and annealed at 420 °C for 8 h.
