# Supplementary material for: Magnetic Properties of SmCo5 + 10 wt% Fe Exchange-Coupled Nanocomposites Produced from Recycled SmCo5
Source: Nanomaterials (Basel). 2020 Jul 3;10(7):1308. doi: 10.3390/nano10071308 (PMC7407525; doi:10.3390/nano10071308)
Supplement: Supplementary file 1 [file nanomaterials-10-01308-s001.pdf]

nanomaterials

Supplementary Information

Magnetic Properties of $SmCo_5$ + 10 wt% Fe Exchange-Coupled Nanocomposites Produced from Recycled $SmCo_5$

Arnab Chakraborty [1,2], Răzvan Hirian [3,*], Gregor Kapun [4] and Viorel Pop [3]

1 Research and Development, MAGNETI Ljubljana d.d., 1000 Ljubljana, Slovenia; arnab.chakraborty@etn-demeter.eu
2 Jožef Stefan International Postgraduate School, 1000 Ljubljana, Slovenia
3 Faculty of Physics, Babeş-Bolyai University, 400084 Cluj-Napoca, Romania; viorel.pop@phys.ubbcluj.ro
4 Department for Material Chemistry, National Institute of Chemistry, 1000 Ljubljana, Slovenia; Gregor.Kapun@ki.si
* Correspondence: Razvan.HIRIAN@ubbonline.ubbcluj.ro

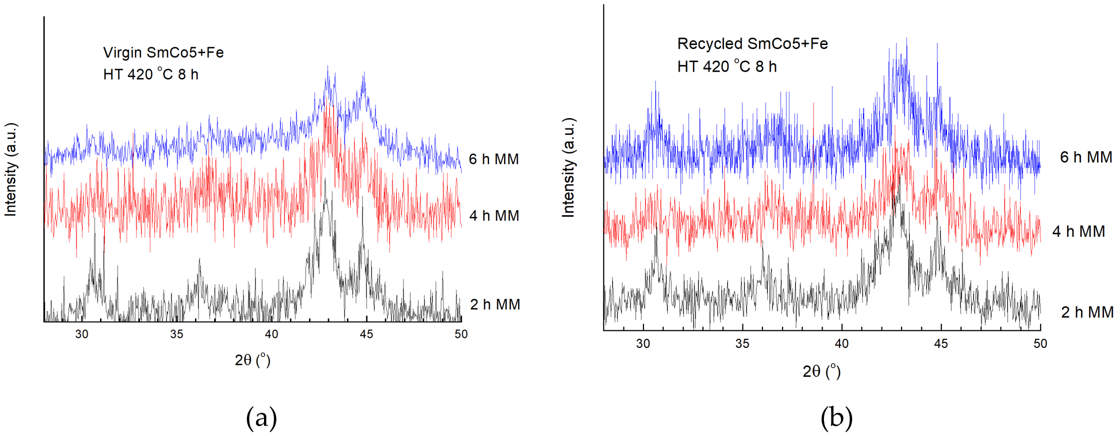

Figure S1. XRD patterns for $SmCo_5$ + 10 wt% Fe magnetic nanocomposite, made using (**a**) virgin or (**b**) recycled $SmCo_5$, milled for 2, 4 and 6 h and annealed at 420 °C for 8 h.

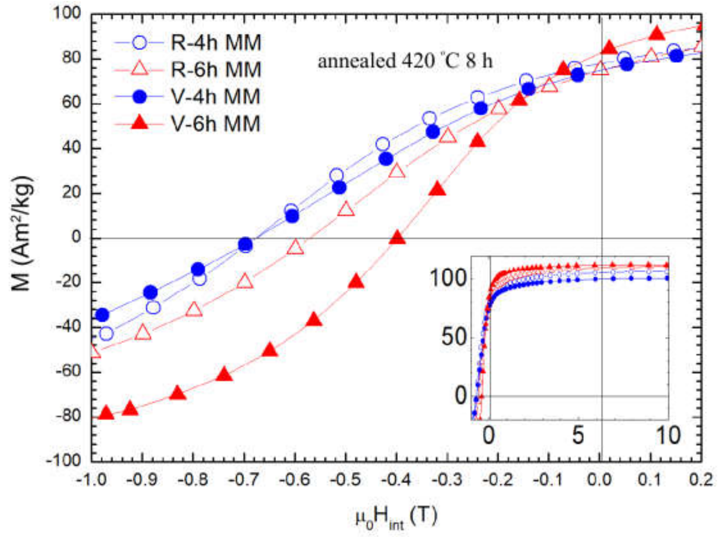

Figure S2. Demagnetization curves (recorded at 300 K) for $SmCo_5$ + 10 wt% Fe magnetic nanocomposite, made using virgin (V) or recycled (R) $SmCo_5$, milled for 4 and 6 h and annealed at 420 °C for 8 h. Magnetization curves for the samples, up to 10 T, are given in figure inset.

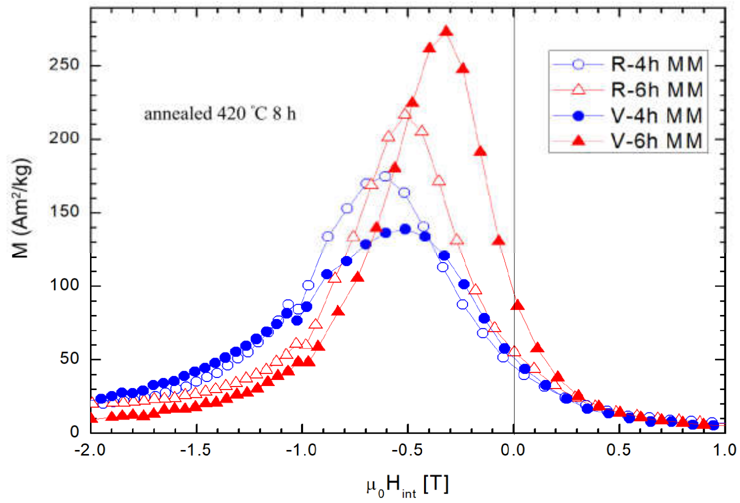

Figure S3. *dM*/*dH* vs *H* plots (recorded at 300 K) for $SmCo_5$ + 10 wt% Fe magnetic nanocomposite, made using virgin (V) or recycled (R) $SmCo_5$, milled for 4 and 6 h and annealed at 420 °C for 8 h.

As seen in Figures 2,3 the magnetic properties of the nanocomposites annealed at 420 °C is much lower than that of the samples annealed at higher temperature (presented in manuscript). Mere strain removal is not enough to ensure good magnetic performance.

© 2020 by the authors. Licensee MDPI, Basel, Switzerland. This article is an open access article distributed under the terms and conditions of the Creative Commons Attribution (CC BY) license (http://creativecommons.org/licenses/by/4.0/).
